# Optimizing image guidance frequency and implications on margins for gynecologic malignancies

**DOI:** 10.1186/1748-717X-8-110

**Published:** 2013-05-02

**Authors:** Carmen Stromberger, Arne Gruen, Waldemar Wlodarczyk, Volker Budach, Christhardt Koehler, Simone Marnitz

**Affiliations:** 1 Department of Radiooncology, Charité Universitätsmedizin Berlin, Campus Virchow Klinikum, Augustenburger Platz 1, Berlin 13353, Germany; 2 Department of Gynecology, Charité Universitätsmedizin Berlin, Berlin, Germany

**Keywords:** Helical tomotherapy, MVCT, Setup accuracy, Gynecological tumors, Image guidance, Margin

## Abstract

**Background:**

To analyze setup deviations using daily megavoltage computed tomography (MVCT) and to evaluate three MVCT frequency reducing protocols for gynecologic cancer patients treated with helical tomotherapy.

**Methods:**

We recorded the setup errors of 56 patients with gynecological cancer observed throughout their whole course by matching their daily MVCT with the planning CT. Systematic and random errors were calculated on a patient and population basis. We defined three different protocols corresponding to MVCTs from the first five fractions (FFF), the first ten fractions (FTF) or from the first and third weeks (505). We compared theoretical. setup errors calculated using these 5 or 10 early MVCT scans with the actual errors found with the remaining fractions to to analyze the residual deviations.

**Results:**

The total systematic (random) deviations had means of −2.0 (3.8)mm, 0.5 (3.4)mm, 0.5 (6.1)mm and −0.5° (0.9°) in vertical (V), longitudinal (LO), lateral (LA), and roll (R) directions, respectively. The proposed three MVCT protocols resulted in minor residual deviations. In all three protocols, 95% of all calculated residual deviations were less than or equal to 5 mm in all 3 directions. When examining the additional minimal CTV-PTV setup margins that were calculated based on these residual deviations, the 505 protocol would have allowed smaller margins than the FFF and FTF protocol, particularly in the V direction.

**Conclusions:**

For patients with gynecologic cancer, the 505-protocol led to the lowest residual deviations and therefore might offer the best approach in reducing the frequency of pre-treatment MVCTs.

## Background

The daily use of image guidance (IGRT) is finding more frequent application in radiation therapy. Modern linear and helical tomotherapy (HT) accelerators are equipped with an onboard imaging device e.g. kV (kilovoltage; cone-beam) CT scanner or MVCT scanner (megavoltage) to localize the target and are capable of applying intensity modulated radiotherapy (IMRT) or volumetric modulated arc therapy (VMAT) to deliver highly conformal dose distributions [1]. HT uses an MVCT imaging tool [2] and has been established for the treatment of patients with gynecologic malignancies [3,4]. For a curative radiotherapy or chemoradiation an adequate treatment of the CTV is essential. The CTV to PTV margin is an established planning method to enable appropriate coverage of the target volume. Pelvic radiotherapy is associated with both an overall higher setup uncertainty in patient positioning and uncertainties due to potential internal organ motion (e.g. uterus, rectum, prostate), thus necessitating wide PTV margins. The shrinkage of the CTV-PTV margin might lead to a reduction of treatment related toxicity (e.g. gastrointestinal) but too narrow margins will increase the risk of inadequate cancer treatment, especially for techniques with highly conformal doses to the target volume. The MVCT of HT allows a daily patient setup verification and correction prior to each treatment, but the scan acquisition, matching with the KV-planning CT and patient repositioning takes significant additional time, depending on the image scan length. This has an impact on patient comfort and tolerance. In this study we investigated the feasibility of reducing the frequency of MVCT scans for patients with gynecological tumors by determining patient and population based systematic and random errors. Three different reduced frequency protocols were analyzed with regard to their respective residual deviations were they to be used in the daily routine.

## Methods

### Patients and patient setup

We analyzed data from a total of 56 patients with cervical cancer, FIGO stage IB-IVA, treated with HT at our institute from June 2008 to December 2009. Pelvic radiotherapy was delivered in 48 cases, 8 patients were treated with pelvic and extended field radiotherapy because of paraaortic lymph node metastases. A total of 34 patients were treated with definitive chemoradiation, 22 patients were treated with an adjuvant radio(chemo)therapy. All patients were placed in a supine position and immobilized using a combiboard with knee-ankle fixation (Unger Medizintechnik, Germany) with their arms folded over their chest. Planning CT scans (CT scanner LightSpeed® from GE Healthcare, General Electric Company, NYSE; GE) were performed at a slice thickness of 3.75 mm.

CTV definition, PTV margins, organ at risks, dose prescription and planning parameters for definitive chemo-radiation with or without paraaortic (extended) field irradiation have been described earlier [3]. For postoperative treatment, the CTV included all regions of potential microscopic disease: the surgical bed, regional lymph node areas (common, external and internal iliacs and the presacral region), and the vaginal cuff. The planning target volume (PTV) was outlined as the CTV plus 1 cm in all directions. The caudal field border was at the obturator foramen, the upper field border was individualized on the basis of the patient’s anatomy to include the common iliac lymph nodes. Patients were initially positioned by aligning skin surface markings with the treatment room’s lasers.

### Treatment verification

MVCTs were typically acquired before each fraction allowing a daily patient setup verification and correction. The scan region and length were defined by the radiation oncologist on the first day of treatment and used for all future scans. The PTV was not routinely scanned over its whole length for patients with a paraaortic extended field. In general, MVCTs were acquired in the normal mode (slice thickness of 4 mm), with a scan length between 15–20 cm. The resulting images were visualized at the HT workstation and the system’s software was used to automatically determine the setup accuracy. A fine resolution matrix (256 × 256) and a variation of mutual information registration called extracted feature fusion based on a mixed “bony and tissue” anatomy were used to rigidly co-register the MVCT images with those from the planning CT, a feature offered by the HT software. Deviations were calculated in the lateral (LA; x axis), longitudinal (LO; y axis), vertical (V; z axis) direction and for rotation in the roll (R; in y axis). Pitch and yaw rotational deviations had to be kept at 0°, since these could not be corrected for by couch or gantry manipulations. Deviations in pitch and yaw rotation were corrected by manually patient repositioning and controlled with a repeat MVCT scan. The initial automatic correction was verified by a radiation oncologist and any necessary corrections were made using bony structures, the soft tissue of the cervical region/tumor (definitive treatment) and PTV localization. Following the registration procedure, the total deviations were corrected for by a shift of the treatment couch in the LO, V and LA directions. The couch used from September 2008 until June 2009 had to be manually adjusted in the LA direction, but was later replaced by a fully automatic couch. Rotational, roll (R) angle was corrected by the fully computerized alteration of the linear accelerator. Radiotherapy was delivered in a mean of 28 fractions (range: 27–31), amounting to a total of 1564 MVCTs.

### Calculation of errors and analysis of imaging protocols

The calculation of the systematic and random setup errors was performed according to the different parameters published by others [5,6].

The total deviation data in the LO, LA, V, and R directions, acquired by daily MVCTs prior to treatment were retrospectively evaluated. These results were used to generate and compare different protocols for the imaging frequencies. The first five fractions (FFF) protocol is calculated on the basis of the patients’ MVCTs acquired on the first five days of radiotherapy. The averages of the LO, LA and V deviations (average total deviation per direction) were calculated and theoretically applied for the subsequent treatments (23 fractions). This protocol resembles the FFF-protocol with five image fractions previously described by Vaandering and colleagues, though this was used for the treatment of the head and neck and brain region [7]. The first ten fractions (FTF) protocol is based and calculated on the patients’ MVCTs acquired during the first ten fractions (days 1–10). The averages of the detected LO, LA and V deviations (average total deviation per direction) were calculated and theoretically applied to the subsequent treatments (18 fractions). The alternate week (505) protocol was based on the 5 MVCTs on days 1–5 and the 5 MVCT scans on days 11–15. The scans for fractions 6–10 were not used for the calculation. The LO, LA and V deviations were then averaged and theoretically applied to the subsequent 13 fractions. For all three protocols, the differences in setup errors in all four degrees of freedom (residual deviations) were calculated by subtracting the averaged total deviations calculated under the specific protocol from the actual deviations detected for the remaining fractions. These residual deviations were then used to determine cumulative distribution functions (CDF). The CDF is a function describing the probability that deviations no greater than the associated deviation (random variable of the CDF) will occur. The steeper the CDF, the smaller the residual deviation and the better the MVCT frequency protocol. The CDFs were determined using built-in functions of Microsoft Excel and used to compare the three different MVCT protocols. Furthermore, we calculated the smallest CTV-PTV margin that is needed if these protocols were applied according to Van Herk’s formula and McKenzie’s IMRT assumption: 2.5 Rres (systematic deviation) + 0.7 rres (random deviation) ensuring that 90% of the patients receive a minimum dose of 95% to the CTV [5,6,8].

### Statistical analysis

Microsoft Excel 2007 and IBM SPSS Version 20 were utilized to calculate the data and to obtain descriptive statistics of the patients’ deviations. Student’s two-sided t-tests were calculated using a significance level of 0.05.

## Results

**Figure 1:**
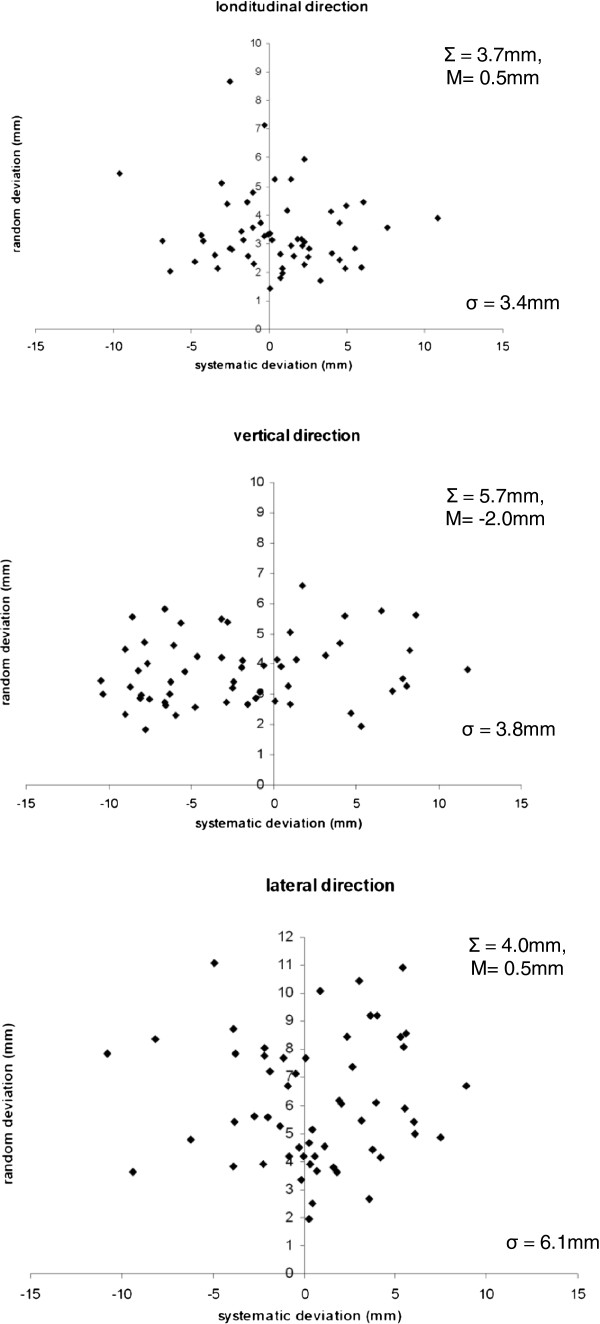
**Total systematic and random deviations for gynecologic patients (n = 56) in the LO, V and LA directions.** Figures include the populations’ mean of all systematic deviations (M), the standards deviations of all systematic deviations (Σ) and the mean of all random deviations (σ).

The systematic and random components of all setup deviations (LO, LA and V directions) were calculated allowing the analysis of the mean systematic deviation (M), the standard deviation of the systematic error (Σ) and the mean random error (σ) for all patients (Table 1, Figure 1). Deviations in roll direction were small (Σ =0.7°, M = −0.5°, σ =0.9°). We did see a negative shift of the patients’ systematic deviations in the V direction due to a known upward pitch of the HT couch. This has been described elsewhere as a 3 mm maximum shift depending on the longitudinal position of the couch [7].

**Table 1:** Systematic and random deviations

	**Systematic deviation (M)**	**Standard deviation of all systematic deviations (Σ)**	**Mean of all random deviations (σ)**
LO	0.5 mm	3.7 mm	3.4 mm
V	−2.0 mm	5.7 mm	3.8 mm
LA	0.5 mm	4.0 mm	6.1 mm

Total systematic and random deviations prior to correction for gynecologic patients (n = 56) in the LO, V and LA directions. Shown are the populations’ mean of all systematic deviations (M), the standards deviations of all systematic deviations (Σ) and the mean of all random deviations (σ).

**Figure 2:**
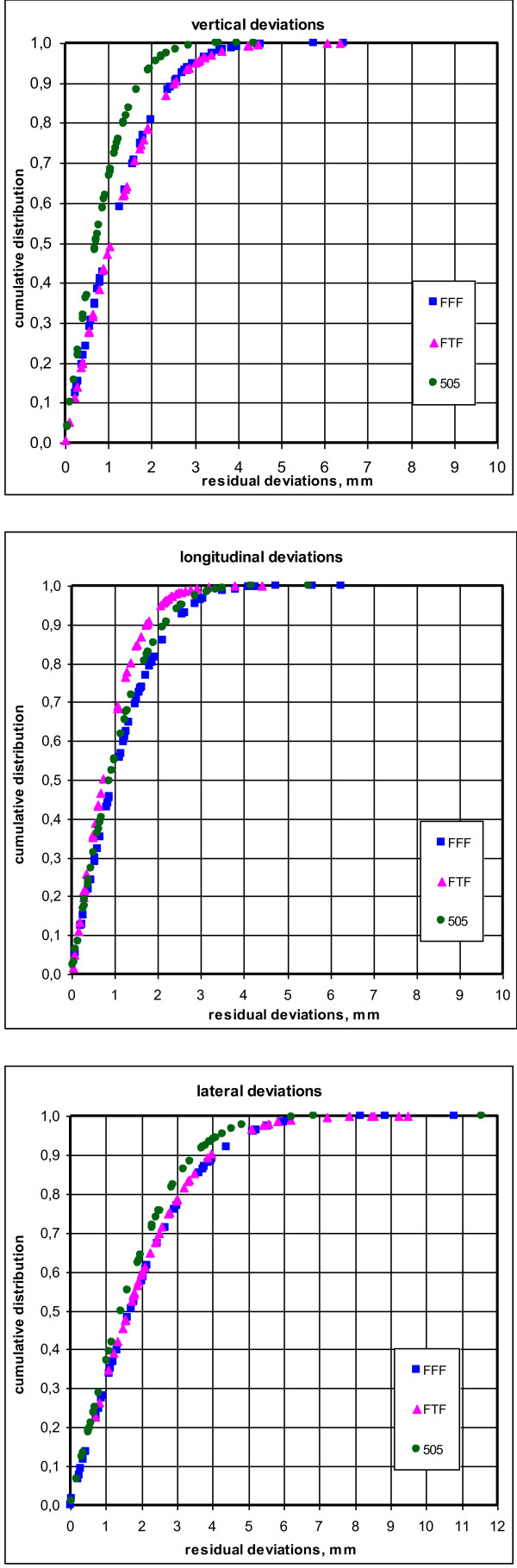
**Residual deviations of MVCT protocols.** Cumulative distribution of the residual deviations in V, LA and LO directions persisting when applying the FFF (blue rectangle), the FTF (pink triangle), the 505 protocol (green dot) for gynecologic tumor patients. p < 0.05 in V and LA for the 505 protocol.

Residual deviations resulting from the application of the three imaging frequency protocols are shown in Figure 2. The 505 protocol resulted in significantly smaller residual deviations in the V and LA direction compared to the FFF (p < 0.001) and FTF (p < 0.02) protocol. No significant difference could be seen in the LO direction. In the FFF and FTF protocol, 5% of the calculated residual vertical deviations were larger than 3 mm compared to 2% for the 505 protocol. In the FFF protocol, 95% of the calculated LO residual deviations were smaller than 4 mm compared to 3 mm for the FTF or 505 protocol, respectively. The residual LA deviations in 95% of the FFF and FTF protocol, were calculated to be smaller or equal to 5 mm compared to 4 mm for the 505 protocol. In all three protocols, 95% of all calculated residual deviations were less than or equal to 5 mm in all 3 directions. When examining the additional minimal CTV-PTV setup margins that were calculated using these residual deviations, the 505 protocol resulted in smaller margins than the FFF and FTF protocol, particularly in the V direction (Table 2).

**Table 2:** CTV-PTV margins

	**CTV-PTV margin**			
	**(2.5 × Rres (systematic deviation) + 0.7 × rres (random deviation due to organ motion & set-up error)**			
	**v (mm)**	**lo (mm)**	**la (mm)**	**r (°)**
**Uncorrected**	14.9	10.2	11.6	1.9
**FFF**	8.4	8.8	13.4	1.7
**FTF**	7.0	6.6	11.5	1.7
**505**	5.5	6.0	9.2	1.4

The calculated minimal CTV-PTV set-up margins for the vertical (V), longitudinal (LO), lateral (LA) and angular (R) deviations for three different MVCT frequency protocols (FFF: first five fractions; FTF: first ten fractions; 505: on alternate weeks: 1st and 3rd week MVCTs).

## Discussion

For patients with gynecologic tumors, HT, conventional IMRT and proton radiotherapy allow normal organ sparing and highly conformal doses to the target volume, with or without an additional simultaneous integrated boost to high-risk areas (e.g. parametria) [3,4,9,10]. An accurate target delineation, highly reproducible patient immobilization, and a clear understanding of internal-organ motion and tumor shrinkage are prerequisites for the optimal use of these sophisticated techniques on such patients [11-13]. The margin definition has a great impact on DVH parameters for the organs at risk in pelvic radiotherapy, especially the small bowel [10,14]. Wide range of different CTV-PTV margins in the treatment of gynecologic malignancies has been published in the literature [15-17]. Up to now, there is no generally accepted standard. Furthermore, to the best of our knowledge, there is not yet sufficient data available to judge the true usefulness of frequent (daily) image guided setup correction [15,18-20] and any impact on margin size or imaging protocols aimed at reducing the frequency of daily imaging for patients with gynecologic malignancies. Techniques and acute toxicities of such treatment have been reported previously [3,9]. A significant inter-fractional (>3 days) improvement of setup accuracy in the V direction (not for LO and LA) has been reported, reflecting a learning curve over time (patient-, technician-wise) has been reported [18]. The use of 3D-imaging over the first few days has been suggested [19].

In order to support - or vary - our institutional routine of using daily MVCTs and a 1 cm CTV to PTV margin in all directions, we retrospective determined the daily setup deviations with respect to three different imaging frequency protocols for 56 gynecologic cancer patients and analyzed their systematic and random components. Our data also suggest a learning curve for patients and/or technicians, since not only the FTF was superior to the FFF protocol but the 505 led to the best result, where indeed the images of fraction 10–15 in addition to the first 5 were used for the calculation of our model. Data based on additional post-treatment MVCT-images seems to indicate that patient motion contributes less than internal organ motion to the whole setup error (1.1 ± 1.3 mm anterior-posterior, -0.3 ± 1.6 mm lateral, 0.2 ± 2.3 mm cranio-caudal) than internal organ motion does [17].

Although there is inconclusive data on uterus/cervix organ motion in the literature, patients setup errors might be reduced to a minimum via daily imaging and corrections [15]. The decrease in setup error as proposed by any of our imaging protocols might help to reduce margins. In all our protocols, 95% of all calculated residual deviations were less than or equal to 5 mm in all 3 directions. Our results showed that the application of the 505 protocol can lead to smaller (<1 cm) CTV-PTV margins at least in areas where organ motion is limited (e.g. vessels/lymph nodes) for definitive and also potentially for postoperative HT, IMRT or VMAT treatment. Not only inter-fractional setup errors but also the intra-fractional movements (organ motion), inter-fractional anatomical changes (bladder- and rectum filling, small bowel movement), potential changes in BMI and tumor shrinkage need to be accounted for. Nevertheless, the uterus/cervical motion, changes in BMI and tumor shrinkage have not been part of our study. However, a study conducted by Collen and colleagues [17] investigated the cervical organ motion in 10 patients with daily MVCTs and found deviations of −3.5 ± 4.9 mm to the left, 0.2 ± 4.5 mm to the right, 0.5 ± 10.1 mm in the anterior, -3 ± 6.9 mm in the posterior, 2.2 ± 8.0 mm in the superior and 0.5 ± 5 mm in the inferior direction, respectively. Uterus motion seems to be even more pronounced than cervical movement [16,17]. Taylor et al. found uterus deviations of 2.7 ± 2.8 mm anterior-posterior, 4.1 ± 4.4 mm superior-inferior and lateral of 0.3 ± 0.8 mm using MR-imaging on two consecutive days and they recommended margins of 15 mm (anterior-posterior), 15 mm (superior-inferior), and 7 mm lateral for the nodal regions and the parametria [16]. Kaatee et al. [21] found a large range of cervical organ movements using a fluoroscopic electronic portal imaging device and radio-opaque markers. The sigma shifts of the markers in the anterior-posterior, superior-inferior, and lateral direction were 3.5, 4.3 and 3.4 mm, respectively. These findings lead them to recommend margins of 10.5, 12.2 and 9.2 mm. Van de Bunt and colleagues [22] used MR-images prior to and after IMRT treatment for cervical cancer patients. The variations in organ movement they found led to propose much larger CTV-PTV margins of 24 mm (anterior), 17 mm (posterior), 11 mm (superior), 8 mm (inferior), 12 mm (right) and 16 mm (left). Santanam et al. [15] recommend a 7 mm CTV-PTV margins in all directions when using daily imaging and daily setup corrections. Stroom et al. [23] proposed a 5 mm CTV-PTV margin based on a study on 14 patients with user defined landmarks (kV, MV orthogonal Portal imaging). Although a CTV-PTV margin of 20/10 mm was used in the study, Lim and colleagues [24] could show that a 5 mm margin might be appropriate for most patients treated with IMRT with the use of a small bowel displacement system if daily setup control is used. The uterus fundus was not part of the CTV in this study. Furthermore, conflicting data exist on the impact of organ filling (bladder, rectum) on cervical movement. Some authors found a correlation between rectum volume/filling and cervical organ motion [16] and/or no correlation with bladder filling [16,25] others observed no correlation of bladder or rectum filling with cervical organ motion [22].

To evaluate the possibility of reducing patients’ “time on machine” by using protocols that allow less frequent imaging but maintaining the higher accuracy offered by IGRT, a further study is required to look into patient comfort via quality of life questionnaires. The practical effect of our protocols on CTV-PTV margins and delivered dose distributions need to be further analyzed.

## Conclusions

This study retrospectively analyzed patient setup deviations for gynecologic cancer patients. Daily positioning and setup correction have improved treatment setup accuracy. We investigated the feasibility of reducing the frequency of pre-treatment MVCTs by calculating the residual deviations that would arise when applying three different imaging frequency protocols. The 505 protocol resulted in significantly smaller residual deviations particularly in the V and LA directions, but not in the LO direction. An analysis of its viability, the global impact on cutting down treatment time and therefore improvements patient comfort are needed to provide more conclusive evidence for a benefit in treatment of gynecologic patients.

### Consent

Written informed consent was obtained from the patient for publication of this report and any accompanying images.

## Competing interests

There are no actual or potential conflicts of interest.

## Authors’ contributions

CS carried out the studies, participated in the design, analyzed and interpreted the data and drafted the manuscript. WW participated in the design of the study, performed the statistical analysis and interpreted the data. AG, VB, CK, SM conceived of the study, and participated in its design and coordination and helped to draft the manuscript. All authors read and approved the final manuscript.
